# Relative Sertoli-cell FSH hyporesponsiveness as a potential contributor to sperm DNA fragmentation: a hypothesis-driven narrative review and proposal of the FERTILE framework

**DOI:** 10.3389/fruro.2026.1915445

**Published:** 2026-08-24

**Authors:** Hussein Kandil

**Affiliations:** Male Reproductive Unit, Fakih IVF Fertility Center, Abu Dhabi, United Arab Emirates

**Keywords:** FERTILE framework, follicle-stimulating hormone, FSH receptor, pharmacogenetics, SDF, Sertoli cell

## Abstract

**Background:**

Sperm DNA fragmentation (SDF) is associated with impaired fertility, adverse reproductive outcomes, and an increased risk of miscarriage. Although oxidative stress is considered the predominant contributor to SDF, accumulating evidence suggests that abnormalities arising during spermatogenesis may also influence sperm DNA integrity. However, whether relative Sertoli-cell FSH hyporesponsiveness contributes to SDF has not been directly investigated.

**Objective:**

To evaluate the biological plausibility that relative Sertoli-cell FSH hyporesponsiveness may contribute to SDF and to propose a multidimensional research framework for future biological investigation.

**Methods:**

A hypothesis-driven narrative review was conducted using experimental, translational, and clinical evidence addressing FSH signaling, Sertoli-cell biology, spermatogenesis, SDF, pharmacogenetics, and FSH-based therapeutic interventions. Relevant literature was identified through structured searches of PubMed, Scopus, and Embase up to June 1, 2026.

**Results:**

Available evidence suggests that circulating FSH concentrations may not accurately reflect effective intratesticular FSH action. Human associative studies, pharmacogenetic findings, and mechanistic experimental evidence collectively support the biological plausibility that inter-individual variation in effective FSH signaling may influence Sertoli-cell support of germ-cell maturation, chromatin remodeling, and cellular quality-control mechanisms. Although direct clinical evidence remains limited, these observations suggest that relative Sertoli-cell FSH hyporesponsiveness could contribute to defective chromatin packaging, persistence of DNA damage, and increased susceptibility to SDF in selected men. To facilitate future investigation of this hypothesis, the FERTILE framework is proposed as a seven-domain conceptual model integrating FSH and effective intratesticular signaling, endocrine markers, receptor and ligand genetics, testicular structure, inflammation and oxidative stress, lifestyle and environmental factors, and evaluation of DNA integrity.

**Conclusions:**

Relative Sertoli-cell FSH hyporesponsiveness represents a biologically plausible but currently unproven contributor to elevated SDF. The proposed FERTILE framework is intended as a conceptual research tool for biological stratification and hypothesis generation rather than a clinical decision-making instrument. Prospective studies incorporating endocrine profiling, pharmacogenetic characterization, standardized SDF assessment, and direct or validated surrogate measures of Sertoli-cell responsiveness are required to determine whether relative Sertoli-cell FSH hyporesponsiveness has independent clinical relevance for sperm DNA integrity and reproductive outcomes.

## 1 Introduction

### 1.1 Definition and clinical relevance of SDF

Sperm DNA Fragmentation (SDF) refers to the presence of single- and/or double-strand DNA breaks within spermatozoa and is increasingly recognized as an important indicator of sperm genomic integrity. It is frequently associated with abnormal chromatin packaging and impaired nuclear integrity. Unlike conventional semen analysis, which evaluates sperm concentration, motility, and morphology, SDF assessment provides complementary information regarding the genomic quality of the male gamete (1–4). Clinical interest in SDF has increased substantially over the past decade and is reflected in recent updates to the WHO Laboratory Manual for the Examination and Processing of Human Semen (5), which recognizes sperm DNA integrity testing as a complementary component of the male infertility evaluation while acknowledging ongoing challenges related to assay standardization and clinical interpretation (2, 6). Elevated SDF has been associated with lower rates of spontaneous conception, an increased risk of miscarriage, particularly among couples with recurrent pregnancy loss (RPL), unexplained infertility, and recurrent assisted reproductive technology (ART) failure (4, 7, 8). Professional society guidelines outline specific clinical scenarios in which SDF testing may provide additional diagnostic information. According to the European Association of Urology (EAU) guidelines, SDF testing should be performed in the assessment of couples with RPL from natural conception, failure of ART, or in men with unexplained infertility (9). For diagnostic purposes, standard karyotype analysis and genetic counseling should be offered to all men with azoospermia or severe oligozoospermia (9). Furthermore, Y-chromosome microdeletion testing should be performed in men with severe oligozoospermia, non-obstructive azoospermia and cryptozoospermia, and considered in selected men with moderately reduced sperm concentrations (9). In men with structural abnormalities of the vas deferens, both the patient and his partner should be tested for cystic fibrosis transmembrane conductance regulator (CFTR) gene mutations (9). Conversely, the American Urological Association (AUA) and American Society for Reproductive Medicine (ASRM) guidelines do not recommend routine SDF testing during the initial evaluation of the infertile couple (10). However, for couples with RPL, the AUA/ASRM guidelines recommend evaluating the male partner with karyotype and SDF (11). In nonazoospermic males with an elevated SDF and persistent infertility, clinicians may consider the utilization of testicular sperm for intracytoplasmic sperm injection (ICSI), as testicular sperm generally exhibit lower levels of DNA damage (10).

### 1.2 Biological origins of SDF

SDF is generally regarded as a multifactorial phenomenon arising from interacting pre-testicular, testicular, and post-testicular influences (2, 12). While oxidative stress has traditionally received the greatest attention as a contributor to sperm DNA damage, increasing evidence suggests that a proportion of DNA fragmentation may originate earlier during spermatogenesis itself, particularly throughout spermiogenesis where extensive chromatin remodeling occurs. The relative contribution of these mechanisms is likely to vary among individuals, emphasizing the biological heterogeneity of SDF. During this phase, histones are initially replaced by transition proteins and subsequently by protamines. This process is accompanied by the formation of transient physiological DNA breaks that facilitate chromatin reorganization. Under normal circumstances, these breaks are efficiently repaired before final chromatin condensation is completed. However, defects in DNA repair, incomplete protamination, abnormal histone retention, or disturbed chromatin packaging may result in persistent DNA damage within mature spermatozoa (13, 14). The molecular path of oxidative sperm DNA decay is driven by the formation of base adducts, particularly 8-hydroxy-2’-deoxyguanosine (8-OHdG), caused by excessive reactive oxygen species (ROS) in the seminiferous or epididymal microenvironment. To repair these damaged bases, sperm utilize the specific repair enzyme 8-oxoguanine glycosylase 1 (OGG1) (1, 12). OGG1 recognizes and excises oxidized guanine bases, generating abasic sites. However, mature spermatozoa lack key downstream components of the base excision repair (BER) pathway, including apurinic/apyrimidinic endonuclease 1 (APE1) and X-ray repair cross-complementing protein 1 (XRCC1), and therefore cannot complete repair of the resulting lesions (15). Repair of residual paternal DNA lesions after fertilization is consequently thought to depend substantially on oocyte-derived DNA-repair machinery (12, 15). Under conditions of excessive oxidative stress, the accumulation of unrepaired oxidative lesions and abasic sites may destabilize sperm DNA and contribute to single- and double-strand breaks (1, 12). If the oocyte’s repair capacity is overwhelmed, particularly in the context of advanced maternal age, the resulting zygote may experience a non-apoptotic block that slows paternal DNA replication, leading to poor embryo development, impaired blastulation, implantation failure, and an increased risk of miscarriage (1, 12). As germ cells progress through differentiation, their intrinsic DNA-repair capacity gradually declines, increasing dependence on the local seminiferous microenvironment to maintain genomic integrity. This highlights the importance of intratesticular quality-control mechanisms that supervise germ-cell development and eliminate defective cells before sperm release (13, 16). Although additional testicular and post-testicular factors such as oxidative stress, varicocele, heat exposure, infection, inflammation, environmental toxicants and adverse lifestyle factors can further induce sperm DNA damage, their impact on intratesticular support mechanisms remains less clearly defined (2, 12, 17). These observations suggest that, in addition to established post-testicular contributors, disturbances within the Sertoli-cell microenvironment may represent another potential mechanism influencing SDF. However, this possibility should be considered within the broader multifactorial pathophysiology of SDF rather than as an alternative or dominant explanation. Taken together, current evidence indicates that SDF represents the final manifestation of multiple interacting biological pathways. Established contributors include oxidative stress, varicocele, infection, inflammation, obesity, metabolic disease, smoking, environmental exposures, and intrinsic defects in spermatogenesis. The present review focuses on one potential intratesticular mechanism involving Sertoli-cell biology and effective FSH signaling, not because it is presumed to be the predominant cause of SDF, but because it remains comparatively underexplored.

### 1.3 Sertoli cells as regulators of germ-cell quality

Sertoli cells are highly specialized somatic cells within the seminiferous epithelium that provide the structural framework and metabolic and paracrine support required for normal spermatogenesis. They participate in multiple stages of germ-cell development by regulating germ-cell adhesion and migration, supplying metabolic substrates, maintaining the seminiferous microenvironment, and orchestrating the apoptotic elimination and phagocytic clearance of defective germ cells (18, 19). These functions become crucial during spermiogenesis, a stage in which chromatin remodeling, cytoplasmic reduction, and sperm release rely on tightly coordinated Sertoli–germ cell interactions (18). Accordingly, Sertoli-cell dysfunction may represent one potential intratesticular mechanism through which sperm with abnormal chromatin packaging or unresolved DNA damage could persist into the ejaculate (13, 18, 19). However, direct evidence linking impaired Sertoli-cell function to increased SDF in humans remains limited, and this relationship should currently be regarded as biologically plausible rather than established. Experimental studies demonstrate that Sertoli cells suppress apoptotic DNA fragmentation in pachytene spermatocytes and round spermatids through paracrine and cell-contact-dependent mechanisms (19), supporting their role in intratesticular quality-control processes. Although these findings provide strong mechanistic support, their direct translation to human SDF requires further investigation. Luteinizing hormone (LH)-dependent testosterone production and follicle-stimulating hormone (FSH) act synergistically to regulate Sertoli-cell function. FSH modulates the expression of genes involved in cell junction integrity, nutrient transport, and metabolic support. Although FSH is not indispensable for the completion of spermatogenesis, experimental and clinical evidence indicates that inadequate FSH stimulation is associated with a substantial reduction in sperm production (20, 21). Whether variation in effective FSH signaling also influences sperm DNA integrity remains the central hypothesis explored in this review.

### 1.4 Why serum FSH may not fully reflect effective FSH action

In the male testis, FSH is a key endocrine regulator of Sertoli-cell function, acting through the FSH receptor (FSHR), a seven-transmembrane G protein-coupled receptor expressed exclusively on Sertoli cells. Upon ligand binding, FSHR couples primarily to Gs protein, stimulating adenylyl cyclase and increasing intracellular cyclic adenosine monophosphate (cAMP), which in turn activates protein kinase A (PKA). In addition to this canonical cAMP/PKA pathway, FSH signaling engages several context-dependent signaling cascades, including ERK/MAPK and PI3K/Akt/mTOR (22, 23). Together, these coordinated pathways regulate Sertoli-cell metabolic activity, paracrine support, junctional dynamics, and cellular survival. In primary Sertoli cells, FSH-induced PKA activation initiates several downstream responses. In proliferating Sertoli cells, FSH activates p70S6K predominantly through PI3K/mTOR-dependent phosphorylation of Thr389, while simultaneously inducing PKA-dependent dephosphorylation of Thr421/Ser424, which exerts an inhibitory modulatory effect on p70S6K activity (24, 25). In differentiated Sertoli cells, Thr389 phosphorylation is constitutively maintained through PI3K/mTOR signaling, whereas FSH primarily enhances p70S6K activity through PKA-dependent dephosphorylation of Thr421/Ser424 (22, 24, 26, 27). This developmental-stage-specific regulation increases p70S6K activity and phosphorylation of downstream targets, including ribosomal protein S6, thereby promoting protein translation. FSH also regulates mTOR complex 1 through PI3K/Akt signaling (22, 23). This pathway regulates Sertoli-cell glucose uptake, glycolysis, and lactate production. Developing spermatids depend substantially on Sertoli-cell-derived lactate for metabolic support. Therefore, disruption of Sertoli-cell metabolic support could theoretically compromise the environment required for late spermatid maturation. Whether this directly affects chromatin-remodeling processes, including histone-to-protamine replacement, has not been established (28, 29). In immature Sertoli cells, FSH-activated PI3K/Akt/mTORC1 signaling promotes proliferation. Separately, connexin-43 is required for normal Sertoli-cell maturation and spermatogenesis and may contribute to blood-testis barrier assembly and the integrity of Sertoli-cell junctional complexes (30). Sertoli cells also play a central role in regulating germ-cell survival and apoptosis. *In vitro*, soluble and contact-dependent Sertoli-cell signals reduced apoptotic DNA fragmentation in pachytene spermatocytes and round spermatids, supporting a role for Sertoli cells in the regulation of germ-cell survival. However, these findings do not directly demonstrate that impaired Sertoli-cell signaling compromises the apoptotic elimination of abnormal germ cells (18, 19, 22). Importantly, the biological activity of FSH cannot be inferred solely from its circulating serum concentration. Inter-individual variation in FSHR expression, receptor polymorphisms, ligand-receptor interactions, receptor trafficking, and downstream signaling efficiency may all influence the biological response to an equivalent circulating FSH concentration (31, 32). Consequently, serum FSH should be regarded as a marker of endocrine stimulation rather than a direct measure of effective biological FSH action at the Sertoli-cell level. Clinical data indicate that some idiopathic infertile men have reduced testicular volume and sperm counts despite subnormal to normal serum FSH levels, suggesting that circulating FSH alone might not accurately reflect intratesticular FSH action (31, 33). Moreover, common polymorphisms in FSHB and FSHR are significantly associated with reproductive hormone levels and testicular volume and are being explored as pharmacogenetic markers that may help predict responsiveness to FSH therapy, particularly in idiopathic infertile men (31, 32). These findings underline the need to consider endocrine markers and pharmacogenetic factors when evaluating effective FSH signaling.

### 1.5 Central hypothesis and scope of this review

Building on these observations, the present review proposes the hypothesis that variation in effective Sertoli-cell responsiveness to FSH may represent one biological modifier of SDF in a subset of infertile men. This hypothesis is based on mechanistic, pharmacogenetic, and indirect clinical evidence rather than direct clinical demonstration. Accordingly, circulating serum FSH concentrations should not necessarily be assumed to reflect effective intratesticular FSH action. In this review, the term “relative Sertoli-cell FSH hyporesponsiveness” refers to reduced effective biological FSH action at the Sertoli-cell level, irrespective of whether this results from alterations in FSH bioactivity, FSH receptor (FSHR) characteristics, or downstream intracellular signaling. Accordingly, the term describes a functional biological phenotype of reduced effective FSH signaling rather than implying that the primary defect resides exclusively within the Sertoli cell or the FSH receptor itself. Consequently, some men with normal or near-normal circulating FSH concentrations may nevertheless exhibit relative Sertoli-cell FSH hyporesponsiveness because of variation in ligand bioactivity, FSHR biology, or downstream signaling competence (31, 32). Such biological variation could reduce the effectiveness of FSH-mediated Sertoli-cell support despite apparently preserved endocrine stimulation. Reduced effective FSH action could theoretically weaken Sertoli-cell support during spermiogenesis, potentially affecting chromatin packaging, coordination of physiological DNA-break repair, regulation of germ-cell survival and apoptosis, and metabolic support (13, 19, 28, 29). These proposed mechanisms are biologically plausible but have not yet been directly demonstrated in human testicular tissue or linked causally to increased SDF. If validated, this model may help explain why some men with apparently preserved endocrine profiles and conventional semen parameters nevertheless exhibit elevated SDF. However, this interpretation should currently be regarded as a testable biological hypothesis rather than an established pathogenic mechanism. The aim of this review is to integrate mechanistic, pharmacogenetic, and clinical evidence relevant to this hypothesis while clearly distinguishing established evidence from biological inference and identifying priorities for future validation studies. To facilitate systematic investigation of this hypothesis, the FERTILE framework is proposed as a qualitative research framework intended to support biological stratification and hypothesis testing. It is not designed as a clinical scoring system or a decision-making tool, and its clinical utility will depend on future experimental and prospective clinical validation. This framework integrates: FSH & effective intratesticular signaling; Endocrine markers, including inhibin B, AMH, and testosterone; Receptor & ligand genetics, including FSHB and FSHR variants and signaling efficiency; Testicular structure, including testicular volume and imaging findings; Inflammation & oxidative stress, including seminal oxidation-reduction potential (ORP), leukocytospermia or pyospermia, genital-tract infection or inflammation, and clinical varicocele; Lifestyle & environmental factors, including BMI and obesity, smoking, alcohol use, dietary habits, heat exposure, environmental toxins, and occupational exposures; and Evaluation of DNA integrity using standardized SDF assays.

## 2 Literature selection method

To identify relevant peer-reviewed literature, a structured search of the PubMed, Scopus, and Embase databases was conducted from database inception through June 1, 2026. Because this article is a hypothesis-driven narrative review, the objective of the literature search was to identify evidence relevant to the proposed biological hypothesis rather than to perform a formal systematic review or meta-analysis. The search strategy employed combinations of Medical Subject Headings (MeSH) and relevant keywords related to SDF, Sertoli-cell function, FSH signaling, and FSH receptor pharmacogenetics. Searches included terms such as “SDF,” “Sperm DNA Damage,” “DNA Fragmentation Index,” “chromatin remodeling,” “protamination,” “Sertoli Cells,” “Sertoli cell function,” “Follicle Stimulating Hormone,” “FSH receptor,” “FSHR,” “pharmacogenetics,” “polymorphism,” and “genotype.” The search prioritized English-language, peer-reviewed primary studies, clinical trials, observational studies, systematic reviews, and meta-analyses. Conference abstracts and non-peer-reviewed preprints were excluded. Editorials, commentaries, and opinion articles were not used as primary evidence but were retained selectively when relevant to conceptual framing or clinical context. Studies were screened and selected by the author according to their relevance to the proposed hypothesis and predefined thematic domains. Preference was given to human clinical studies, systematic reviews, meta-analyses, and translational studies where available, while animal and *in vitro* studies were included primarily to support mechanistic discussion when direct human evidence was unavailable. Retrieved evidence was evaluated for its relevance to Sertoli-cell biology, FSH signaling, SDF, reproductive endocrinology, and pharmacogenetic mechanisms. Accordingly, direct clinical evidence, associative human evidence, mechanistic experimental findings, and hypothesis-generating inferences are presented separately throughout the review to clearly distinguish established knowledge from biological interpretation. Where direct human evidence is lacking, mechanistic interpretations are presented explicitly as biological hypotheses requiring future validation. To facilitate interpretation of the available literature, the retrieved evidence was subsequently organized into four categories: Direct Clinical Evidence (randomized and prospective studies evaluating exogenous FSH interventions), Associative Human Evidence (observational studies examining FSH-related genetic and phenotypic associations), Mechanistic Experimental Evidence (*in vitro*, ex vivo, and animal studies investigating Sertoli-cell signaling pathways), and Integrative Inference (narrative synthesis of mechanistic, translational, and clinical evidence used to generate biological hypotheses). Because this was a hypothesis-driven narrative review, no formal risk-of-bias assessment or quantitative meta-analysis was performed.

## 3 Evidence hierarchy and synthesis

The available evidence supporting a relationship between effective FSH signaling and SDF varies considerably in both design and strength. Accordingly, this section distinguishes direct clinical evidence, associative human observations, mechanistic experimental studies, and integrative biological inferences. This organization is intended to clearly differentiate established findings from hypothesis-generating interpretations and to provide the context in which the proposed conceptual model should be interpreted.

### 3.1 Direct clinical evidence

Direct human clinical evidence relevant to the relationship between effective FSH signaling and SDF is derived primarily from prospective clinical studies, including controlled and pharmacogenetic investigations evaluating FSH therapy in infertile men. Several trials have reported improvements in selected semen parameters and reductions in SDF after exogenous FSH treatment, particularly in subgroups stratified by baseline endocrine or genetic profiles (34, 35). The most relevant pharmacogenetic study demonstrated that recombinant FSH reduced SDF in idiopathic infertile men depending on the FSHR p.N680S genotype, with a significant reduction in the DNA fragmentation index among homozygous N carriers (34). Other small studies have reported variable effects of FSH on SDF, but differences in patient selection, baseline gonadotropin levels, FSH preparation, dose, treatment duration, SDF assay, and reproductive endpoints limit comparability (35). Overall, although the available studies provide the strongest direct clinical evidence linking recombinant FSH therapy with improvements in SDF, they remain heterogeneous with respect to patient selection, treatment protocols, FSH preparations, treatment duration, and SDF assessment methods. Accordingly, the findings support the biological plausibility of a relationship between FSH signaling and sperm DNA integrity, but they do not establish that relative Sertoli-cell FSH hyporesponsiveness is the underlying cause of increased SDF or support routine clinical application without further patient stratification and prospective validation (35, 36). Instead, they provide the clinical rationale for future studies designed to directly test this hypothesis. **Table 1** summarizes the principal clinical studies evaluating recombinant FSH therapy and its reported effects on SDF.

**Table 1:** Clinical evidence summary of exogenous FSH therapy in men with elevated SDF.

Study Reference	Study design & cohort size (n)	Patient stratification method	SDF assay & baseline threshold	FSH regimen	Major findings & limitations
Simoni et al., 2016 (34)	Multicenter, prospective pharmacogenetic trial; 66 men with DFI data (55 completed)	Stratified by FSHR p.N680S genotype (N/N vs S/S), with additional post-hoc stratification by sperm count and FSHB −211G>T.	TUNEL; elevated baseline SDF	rFSH 150 IU s.c. every other day for 12 weeks	DFI reduction seen only in FSHR N/N men, driven by viable sperm; Conventional semen parameters were heterogeneous and were not primary endpoints. Small, uncontrolled, single-regimen study with no pregnancy endpoints.
Muratori et al., 2018 (35)	Narrative review; synthesizes six FSH–SDF clinical trials (383 treated men) plus mechanistic data.	Summarizes trial-specific stratifications (idiopathic OAT vs broader abnormal semen, baseline SDF, limited pharmacogenetics).	Various assays (TUNEL, SCSA, SCD) cut-offs and inclusion thresholds heterogeneous and often absent.	Mixed regimens (recombinant vs. urinary FSH) typically 75–150 IU daily or q48h for ~3 months	Overall modest SDF reduction with FSH; semen parameter changes inconsistent. Marked heterogeneity in assays, cut-offs, regimens, and endpoints; calls for standardized, pharmacogenetically informed trials.
Colacurci et al., 2018 (72)	Phase II multicenter prospective open-label trial; 103 men with evaluable DFI.	Idiopathic/unexplained infertile men with FSH 1–8 IU/L and reduced sperm number/motility; post-hoc subgroups by baseline DFI <17 vs ≥17, smoking, and FSH quartiles.	TUNEL microscopy; no predefined cut-off, median DFI 17% used for subgrouping	rFSH 150 IU s.c. every other day for 3 months	Mean DFI fell ~35%, especially in men with baseline DFI <17% and FSH 2.16–4.27 IU/L; sperm concentration, total count, motility, and morphology also improved. Single-arm, no control, arbitrary DFI threshold, no pregnancy outcomes.
Lispi et al., 2022 (60)	Retrospective pooled IPD analysis of three rFSH trials; 251 idiopathic infertile men.	DFI responders (≥20% relative DFI decrease) vs non-responders; analyses by age, BMI, and hormone levels, especially testosterone.	Various assays inherited from the three source trials; responders defined *post hoc* as ≥20% relative DFI reduction.	rFSH 150 IU s.c. every other day for 3 months in all included trials.	Overall ~20% DFI reduction; higher post-treatment testosterone and younger age associated with greater DFI improvement. Pooled secondary analysis, heterogeneous source trials, no unified untreated control, no pooled pregnancy endpoints.
Ayati et al., 2023 (73)	Retrospective observational before–after study in a single andrology clinic; infertile men with male-factor infertility and elevated DFI; n = 94 (age 27–50 years).	All included if DFI > 20% before treatment and met exclusion criteria (no azoospermia, varicocele, cryptorchidism, endocrine hypogonadism, karyotype/Y-microdeletion, chemo/radiotherapy). No internal control arm; everyone received combination therapy.	SDF assessed with SDFA (halo) kit (Dain Bioassay, Iran); ≥200 sperm scored per slide; inclusion required DFI > 20%; baseline median DFI 32.5% (IQR 17.0).	Recombinant FSH 75 IU s.c. every 5 days for 2 months, combined with daily oral antioxidants (selenium 60 μg, vitamin E 200 IU, CoQ10 100 mg, folic acid 5 mg).	Median DFI decreased from 32.5% to 15.0% after 2 months (mean relative reduction 50.16%, p ≤ 0.001). Conventional semen parameters showed no significant changes.

### 3.2 Associative human evidence

Unlike the direct clinical studies discussed above, the evidence summarized in this subsection is primarily associative and does not directly establish a causal relationship between effective FSH signaling and SDF. Instead, these studies provide indirect human evidence suggesting that inter-individual variation in endocrine function, pharmacogenetics, and Sertoli-cell biology may influence spermatogenesis and sperm DNA integrity. Several studies have investigated how endogenous FSH levels and FSHB/FSHR polymorphisms relate to male reproductive traits. Common FSHR variants, including the linked Thr307Ala/Asn680Ser exon 10 variants and the promoter −29G>A variant, together with the FSHB promoter −211G>T variant, have been associated with modest differences in serum FSH, inhibin B, and testicular volume, with less consistent associations reported for sperm concentration (31–33). Although these polymorphisms may modify testicular responsiveness to FSH, they should be interpreted as biological modifiers rather than direct evidence of relative Sertoli-cell FSH hyporesponsiveness or increased SDF. Furthermore, observational studies suggest that specific FSHB/FSHR genotypes modulate the efficiency of FSH signaling in the testis, offering a biologically plausible explanation for why some men with low-normal FSH levels exhibit impaired spermatogenesis and potentially increased susceptibility to SDF. This hypothesis is biologically plausible based on evidence that FSH withdrawal increases germ-cell DNA fragmentation and that defects in chromatin remodeling and DNA repair during spermiogenesis can result in persistent DNA damage (13, 16, 31, 33, 37, 38). However, these genotype–phenotype associations are modest, not consistently replicated across populations, and do not establish causality. In parallel, endocrine biomarkers such as inhibin B and anti-Müllerian hormone (AMH) correlate with spermatogenic output and provide indirect information regarding Sertoli-cell function. However, they are neither specific indicators of SDF nor direct measures of effective biological FSH responsiveness (39, 40). Collectively, the available associative human evidence supports the biological plausibility that inter-individual variation in effective FSH signaling may influence testicular function. That said, these observations remain indirect and should not be interpreted as establishing a causal relationship between relative Sertoli-cell FSH hyporesponsiveness and increased SDF.

### 3.3 Mechanistic experimental evidence

The evidence summarized in this subsection is derived predominantly from experimental animal models, ex vivo preparations, and *in vitro* studies. Although these investigations provide important mechanistic insights into the biological actions of FSH within the seminiferous epithelium, they should not be interpreted as direct evidence that the same mechanisms operate in humans or directly contribute to SDF. Experimental *in vitro* and animal models have been used to investigate FSH-dependent mechanisms regulating Sertoli-cell biological function, as well as the consequences of FSH deprivation. FSH suppression or immunoneutralization has been associated with increased germ-cell apoptosis in animal models, whereas FSH withdrawal increased DNA fragmentation in primary spermatocytes and spermatids in cultured human seminiferous tubules (16, 20). These experimental observations support the biological plausibility that impaired effective FSH signaling could compromise germ-cell quality control. However, direct confirmation of these broader signaling mechanisms in human Sertoli cells and their relationship to sperm DNA integrity remains limited. Experimental studies, conducted predominantly in immature animal Sertoli-cell models, indicate that FSH/FSHR signaling engages multiple intracellular cascades, including cAMP/PKA, ERK1/2, and PI3K/Akt/mTORC1 pathways (22, 23). These pathways regulate Sertoli-cell proliferation, differentiation, metabolism, and junctional function. FSH may also modulate retinoic acid metabolism and signaling, thereby influencing spermatogonial differentiation and meiotic entry (41). Collectively, these signaling pathways provide a coherent biological framework through which FSH may regulate Sertoli-cell support of spermatogenesis. However, their contribution to sperm DNA integrity in humans remains largely inferential, and the proposed relationship between relative Sertoli-cell FSH hyporesponsiveness and increased SDF requires confirmation in prospective human studies.

### 3.4 Integrative inference: a conceptual biological model

The following section integrates the direct clinical, associative human, and mechanistic experimental evidence discussed above into a conceptual model. The mechanisms proposed below should be interpreted as hypothesis-generating biological inferences rather than experimentally established pathways linking relative Sertoli-cell FSH hyporesponsiveness to increased SDF in humans. The central hypothesis of this review is that relative Sertoli-cell FSH hyporesponsiveness may produce multiple small but cumulative disturbances during spermiogenesis rather than a single isolated defect. Although each component of the proposed model is supported to varying degrees by experimental or clinical observations, their integration into a unified biological framework remains conceptual and has not yet been validated in human studies. Reduced effective FSH signaling could theoretically alter the timing and expression of Sertoli-cell support factors involved in transition protein and protamine regulation, retinoic acid metabolism, antioxidant defense, metabolic substrate delivery, and paracrine signaling between Sertoli cells and germ cells. Incomplete histone-to-protamine exchange could result in under-protaminated sperm chromatin with reduced compaction, consistent with the “defective maturation” model in which incompletely condensed chromatin may be more susceptible to oxidative injury (12, 14). Whether impaired effective FSH signaling directly alters chromatin packaging in human sperm remains unknown. Coordination of physiological DNA-break repair during chromatin remodeling may be compromised, allowing normally transient topo II–mediated breaks to persist as unrepaired lesions (13, 14). Direct evidence linking reduced effective FSH signaling to defective DNA repair in human spermatogenesis is currently lacking. Apoptotic culling and Sertoli-cell-mediated control of defective germ cells may be weakened, permitting some cells with sublethal chromatin defects to escape elimination and continue through spermiogenesis (13, 16, 42, 43). This mechanism is inferred primarily from experimental evidence and has not been directly demonstrated in human infertility. Finally, reduced Sertoli-cell metabolic support, including impaired lactate supply and substrate handling, may compromise germ-cell viability, while reduced antioxidant protection within the male reproductive tract may increase the susceptibility of vulnerable sperm to oxidative DNA damage (12, 28, 29). The potential contribution of impaired Sertoli-cell metabolic support to SDF therefore remains hypothetical. These observations provide a biologically coherent conceptual framework through which variation in effective FSH signaling could influence sperm DNA integrity. However, the proposed model remains inferential because it integrates evidence derived from different experimental systems rather than direct human studies. Prospective investigations incorporating functional assessment of Sertoli-cell responsiveness, pharmacogenetic profiling, and standardized SDF testing will be required to validate or refute this hypothesis.

### 3.5 Assessment and measurement of SDF

SDF assays provide a functional assessment of sperm DNA integrity but do not identify the underlying biological mechanism responsible for DNA damage. Consequently, abnormal SDF results should be interpreted within the broader clinical context and in conjunction with conventional clinical, endocrine, and reproductive findings. Several laboratory methods are currently available to assess SDF, including the terminal deoxynucleotidyl transferase dUTP nick-end labeling (TUNEL) assay, sperm chromatin structure assay (SCSA), sperm chromatin dispersion (SCD) assay, and comet assay. Each technique is based on a different physicochemical principle and measures different aspects of sperm DNA damage, thereby generating distinct numerical values and assay-specific thresholds. Because these assays evaluate distinct characteristics of sperm DNA integrity, they should not be considered interchangeable. Consequently, quantitative SDF values and cut-offs established for one method cannot be directly extrapolated to, or compared with, those obtained using another assay. This methodological variability remains one of the major obstacles to standardization of SDF testing in both research and routine clinical practice (2, 44). Within the context of the present hypothesis, elevated SDF should therefore be regarded as a downstream biological phenotype rather than direct evidence of impaired effective FSH signaling. Accordingly, SDF testing should be viewed as one component of a comprehensive male infertility evaluation. Although it may identify patients with increased sperm DNA damage, additional clinical, endocrine, genetic, and functional biomarkers will be required to determine whether relative Sertoli-cell FSH hyporesponsiveness contributes to the observed phenotype.

### 3.6 Clinical interpretation of SDF testing

The clinical interpretation of SDF requires consideration of its multifactorial etiology. Elevated SDF reflects a downstream biological phenotype that may arise from multiple pre-testicular, testicular, and post-testicular mechanisms. Accordingly, interpretation of SDF should always be integrated with clinical findings, endocrine evaluation, genetic factors, and established causes of male infertility. Higher SDF levels have been associated with reduced fertility potential, unexplained or idiopathic infertility, RPL, and poorer ART outcomes, although the association appears more consistent for IVF than for ICSI (1, 7, 8). Within this context, the present hypothesis proposes that relative Sertoli-cell FSH hyporesponsiveness may represent one additional biological contributor in a subset of patients rather than a universal explanation for elevated SDF. At present, however, no validated clinical biomarker or functional assay is available to directly identify relative Sertoli-cell FSH hyporesponsiveness in individual patients. Consequently, its proposed relationship with elevated SDF should currently be regarded as a biologically plausible but unvalidated hypothesis. Nevertheless, SDF is not an unequivocal biomarker of fertility potential but a variable measure of the degree of DNA damage, which can vary greatly depending on several factors, including the aging process, lifestyle, environmental exposures, and other male infertility markers. Increased oxidative stress, for instance, stemming from smoking habits, obesity, varicocele, or infection can result in higher SDF values (2, 3, 12). Importantly, modest elevations in SDF do not inevitably lead to poor reproductive outcomes, particularly when oocyte DNA-repair capacity is sufficient to correct paternal lesions (1, 12, 45). Elevated SDF may adversely affect embryo development and pregnancy outcomes and may be present even when conventional semen parameters are within normal ranges (1, 2, 45). Accordingly, clinicians should interpret SDF results using a holistic approach that integrates conventional semen analysis, endocrine evaluation, genetic testing, female-related factors, and the broader clinical context. Current professional society guidelines support SDF testing in selected clinical scenarios, particularly unexplained infertility, RPL, and ART failure, while not recommending its routine use in the initial evaluation of all infertile men (9, 10). Within the context of the present hypothesis, interpretation of elevated SDF may also benefit from consideration of intratesticular mechanisms such as Sertoli-cell function and effective FSH signaling. The Global Andrology Forum (GAF) 2026 guidelines on antioxidant use in male infertility were developed through a modified Delphi panel of 151 international experts, followed by a GRADE assessment involving 84 experienced physicians. The guidelines conclude that a complete clinical and andrological evaluation should precede antioxidant therapy. They emphasize documenting oxidative stress, assessing underlying causes, and avoiding indiscriminate or prolonged antioxidant use. Lifestyle modifications, particularly smoking cessation and weight loss, are strongly recommended and may improve semen parameters in men with idiopathic infertility or oxidative stress-related risk factors (46). Overall, the GAF 2026 consensus supports antioxidant use in selected men with idiopathic infertility, elevated SDF, or oxidative-stress risk factors, but frames these as conditional, individualized recommendations rather than routine or universal therapy, and specifically warns against indiscriminate or prolonged antioxidant use (46). Accordingly, the clinical value of the present hypothesis lies not in providing an immediate diagnostic tool, but in offering a biologically plausible framework that may guide future biomarker discovery, patient stratification, and prospective interventional studies.

## 4 Mechanistic pathways linking reduced effective FSH action to elevated SDF

The pathways discussed in this section represent biologically plausible mechanisms through which reduced effective FSH signaling could theoretically contribute to SDF. They are derived from integration of mechanistic, translational, and limited clinical evidence and should therefore be interpreted as hypothesis-generating rather than experimentally validated mechanisms in humans. Each pathway is discussed separately for conceptual clarity, although these mechanisms are likely to interact within the seminiferous epithelium.

### 4.1 Defective chromatin packaging

During spermiogenesis, histones are progressively replaced by transition proteins and protamines, resulting in highly compacted and transcriptionally silent sperm chromatin. Under-protaminated sperm chromatin is less compact and may be more vulnerable to oxidative damage, and protamine deficiency is associated with increased SDF and reduced male fertility (12, 14). FSH-mediated Sertoli-cell support may indirectly influence chromatin remodeling by maintaining the metabolic, paracrine, and developmental environment required for germ-cell maturation and the histone-to-protamine transition (28, 29). Evidence linking transition-protein and protamine defects, together with abnormal histone retention, to compromised sperm nuclear integrity underscores the potential importance of this pathway in SDF biology (13, 14). However, a direct causal relationship between impaired FSH signaling and incomplete protamination in humans has not been demonstrated and remains a biologically plausible hypothesis requiring prospective validation.

### 4.2 Impaired DNA-repair coordination

The repair of physiological DNA strand breaks during chromatin remodeling is thought to depend on coordinated chromatin remodeling, transition-protein dynamics, protamine incorporation, and DNA repair mechanisms operating during spermiogenesis. These processes occur within a Sertoli-cell-regulated seminiferous microenvironment that provides metabolic, developmental, and paracrine support for germ-cell maturation (22, 28, 29). FSH-dependent pathways such as cAMP/PKA and PI3K/Akt/mTOR signaling regulate Sertoli-cell proliferation, metabolism, differentiation, and functional activity, thereby shaping the intratesticular microenvironment that supports germ-cell development and maturation (22, 28). Relative Sertoli-cell FSH hyporesponsiveness may diminish these supportive functions, creating a less favorable environment for the completion of DNA repair. Theoretically, transient DNA breaks generated during normal spermiogenesis may persist as unrepaired lesions in mature spermatozoa if chromatin remodeling or DNA-repair processes are disrupted (13). Although biologically plausible, the contribution of this pathway to human SDF remains hypothetical and requires prospective validation.

### 4.3 Weak apoptotic culling of damaged germ cells

Sertoli cells regulate the balance between germ-cell survival and apoptosis through paracrine factors and cell-contact-dependent signals. They also phagocytose apoptotic spermatogenic cells (47). Relative Sertoli-cell FSH hyporesponsiveness could theoretically disturb survival-apoptosis signaling and Sertoli-cell-mediated germ-cell quality control. However, whether reduced FSH action impairs the recognition or phagocytic clearance of defective germ cells has not been established. FSH withdrawal increased DNA fragmentation in primary spermatocytes and spermatids in cultured human seminiferous tubules, supporting a role for FSH-dependent Sertoli-cell signaling in germ-cell survival and DNA integrity (16). Whether such alterations in Sertoli-cell-mediated quality control contribute directly to increased SDF in humans remains unknown.

### 4.4 Metabolic and paracrine microenvironmental disruption

Sertoli-cell support depends on metabolic and secretory functions, including lactate production, growth-factor and cytokine signaling, and cellular metabolic homeostasis, several of which are regulated directly or indirectly by FSH (22, 28, 29). The importance of Sertoli-cell lactate metabolism for the metabolic environment supporting spermatogenesis is illustrated by findings that conditional deletion of Ldha in Sertoli cells altered 2,456 metabolites in sperm and resulted in profound defects in spermiogenesis (48). Clinical and mechanistic evidence linking metabolic and oxidative disturbances to increased SDF suggests that such changes may exacerbate pre-existing chromatin defects, increase oxidative susceptibility, and favor the persistence of spermatozoa with damaged DNA (2, 12). Within the proposed conceptual model, metabolic and paracrine disruption may represent a permissive mechanism that could amplify the consequences of defective chromatin remodeling and impaired germ-cell quality control.

## 5 Translational implications and candidate biomarkers

The proposed hypothesis also has potential translational implications by identifying candidate biomarkers that may facilitate assessment of effective FSH signaling and its downstream biological consequences. At present, however, no validated biomarker or functional assay can directly identify relative Sertoli-cell FSH hyporesponsiveness in clinical practice. The candidate biomarkers discussed below should therefore be regarded as complementary research tools that may improve biological stratification and facilitate future validation of the proposed hypothesis, rather than as established diagnostic tests.

### 5.1 FSHB and FSHR genotyping

Studies suggest that polymorphisms involving genes encoding the FSH β-subunit (FSHB) and the FSHR may influence serum FSH concentrations, inhibin B, testicular volume, selected semen parameters, and clinical response to FSH therapy (32–34). For example, the FSHB −211G>T promoter variant may alter FSH production, while FSHR variants such as −29G>A, Thr307Ala, and Asn680Ser may affect receptor expression, structure, or signaling responsiveness (31, 32). Furthermore, from a pharmacogenetic perspective, it is suggested that selected FSHR genotypes may identify subgroups more likely to demonstrate SDF improvement following FSH therapy (34). However, these pharmacogenetic associations have not been validated as surrogate markers of relative Sertoli-cell FSH hyporesponsiveness. These genotype-based associations provide supportive evidence but must be interpreted cautiously. They represent individual genetic contributors and do not validate the composite clinical framework. At present, FSHB and FSHR genotyping should be regarded as tools for biological stratification and hypothesis-driven research rather than as diagnostic markers of SDF or relative Sertoli-cell FSH hyporesponsiveness (31, 36).

### 5.2 Receptor expression and downstream signaling readouts

In addition to genetic profiling, assessment of FSHR expression and downstream signaling could theoretically provide a more direct evaluation of effective FSH signaling at the Sertoli-cell level. Potential functional readouts of FSHR activation include cAMP accumulation, cAMP response element-binding protein (CREB) phosphorylation, ERK1/2 activation, PI3K/Akt/mTORC1 signaling, and activation of p70S6K, all of which have been documented in experimental Sertoli- and granulosa-cell models of FSH signaling (22, 49). Although these signaling readouts have been extensively characterized in experimental models, their clinical applicability as biomarkers of relative Sertoli-cell FSH hyporesponsiveness remains to be established. The measurement of these markers in testicular biopsy specimens, ex vivo seminiferous tubule cultures, or Sertoli-cell-enriched experimental systems could theoretically provide insights into individual FSH responsiveness (22). Such functional assays remain research tools because they require invasive tissue sampling and specialized laboratory techniques. Their future clinical utility will depend on the development and validation of less invasive surrogate biomarkers capable of reflecting effective intratesticular FSH signaling. Candidate non-invasive proxies include seminal-plasma proteomic signatures, testis-derived proteins detectable in seminal plasma, and seminal extracellular-vesicle-associated microRNAs. These candidates remain indirect research tools rather than validated measures of Sertoli-cell FSH responsiveness and are discussed in greater detail in Section 5.4 (50–52).

### 5.3 Endocrine markers of Sertoli-cell function

Among endocrine markers of Sertoli-cell function, inhibin B is the principal serum marker used in adult male reproductive medicine, reflecting both Sertoli-cell function and the degree of spermatogenic activity while participating in the negative feedback mechanism involving pituitary FSH (39). In contrast, AMH is secreted at high levels by immature Sertoli cells during fetal life and childhood, is primarily informative for assessing testicular and Sertoli-cell function in prepubertal and early pubertal boys, and declines during puberty as Sertoli cells mature under androgen influence, remaining relatively low in adulthood (53–56). The combined interpretation of inhibin B and FSH has been proposed to improve the assessment of Sertoli-cell performance, but its specific relationship to SDF is unclear (39, 40). Furthermore, although these endocrine markers provide indirect information regarding Sertoli-cell function, their ability to identify patients with impaired effective FSH signaling has not been established. Endocrine markers may support biological phenotyping but should not be interpreted as direct indicators of relative Sertoli-cell FSH hyporesponsiveness or as predictors of SDF.

### 5.4 Transcriptomic, microRNA, and proteomic candidates

High-throughput profiling has revealed a wide range of Sertoli-cell-enriched genes involved in metabolism, junctional regulation, and paracrine signaling (57). Since disruption of FSHR signaling can remodel Sertoli-cell transcriptional programs, transcriptomic readouts may provide indirect evidence of altered effective FSH signaling (58). Seminal plasma microRNAs and proteins may provide indirect information about testicular function, oxidative stress, apoptosis, and sperm maturation (50–52). These markers are attractive because they could allow non-invasive assessment of pathways related to Sertoli-cell function, spermatogenesis, oxidative stress, apoptosis, and sperm quality. However, their specificity for effective FSH signaling remains uncertain, and the available data are largely associative and heterogeneous. Transcriptomic, microRNA, and proteomic markers should therefore be regarded as research tools for mechanistic discovery, biological stratification, and future biomarker development rather than as clinically validated biomarkers.

### 5.5 Integration with SDF testing

To establish a biologically informed framework for stratifying infertile men, SDF results could be integrated with endocrine, genetic, and molecular profiling. Within a prospective clinical research framework, men with elevated SDF could be prospectively classified according to FSHB and FSHR genotype, serum FSH, inhibin B, AMH, testosterone, testicular volume, conventional semen parameters, and relevant clinical confounders such as varicocele, infection, leukocytospermia, obesity, heat exposure, smoking, and oxidative stress (12, 31, 35). This approach may help identify biologically distinct subgroups of men with elevated SDF, including those with features suggestive of impaired effective FSH signaling and others in whom SDF may be more strongly associated with post-testicular mechanisms. Such stratification could improve biological phenotyping and facilitate selection of patients most likely to benefit from FSH-based therapy while avoiding unnecessary interventions in others (36). Nevertheless, current evidence does not justify relying on any single biomarker, whether serum FSH, inhibin B, AMH, FSHB/FSHR genotype, or SDF result, as a definitive indicator of Sertoli-cell FSH hyporesponsiveness (31, 36). At present, the most defensible application of these markers is within prospective research models designed to determine whether measures of effective FSH signaling are associated with SDF severity or predict treatment response.

### 5.6 FERTILE framework: implementation and interpretation

**Figure 1:**
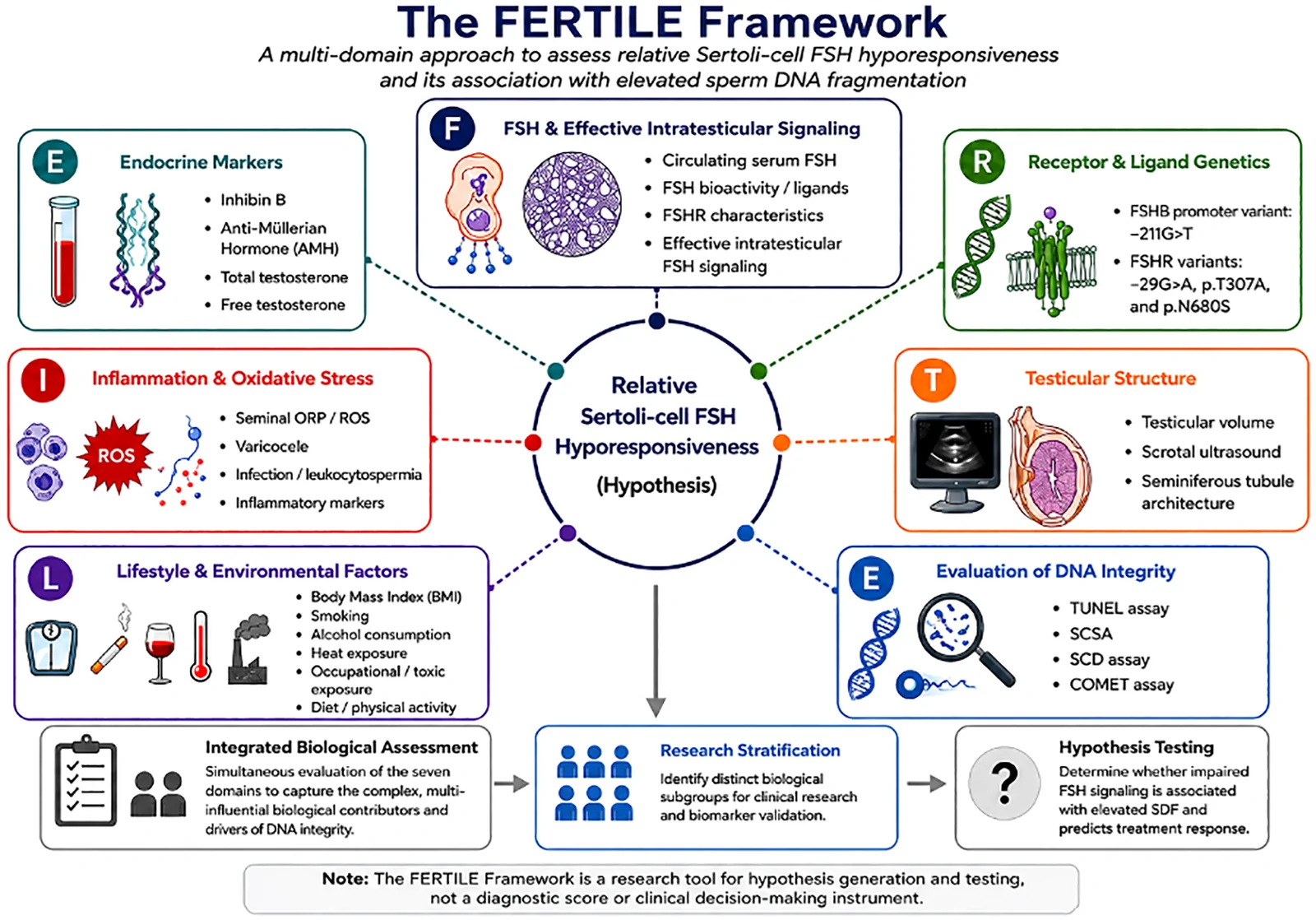
The FERTILE framework for investigating relative Sertoli-cell FSH hyporesponsiveness and sperm DNA fragmentation. The FERTILE framework is a seven-domain conceptual model integrating F, FSH and effective intratesticular signaling; E, endocrine markers; R, receptor and ligand genetics; T, testicular structure; I, inflammation and oxidative stress; L, lifestyle and environmental factors; and E, evaluation of DNA integrity. The framework is intended to support biological stratification, hypothesis generation, and the design of prospective translational studies examining whether variation in effective Sertoli-cell responsiveness to FSH may contribute to sperm DNA fragmentation. It is a qualitative research model and should not be interpreted as a validated clinical score, diagnostic instrument, or treatment decision-making tool. The relative contribution of each domain may vary among individuals, and abnormalities within any single domain do not establish relative Sertoli-cell FSH hyporesponsiveness. FSH, follicle-stimulating hormone; FSHB, follicle-stimulating hormone beta subunit; FSHR, follicle-stimulating hormone receptor; AMH, anti-Müllerian hormone; ORP, oxidation–reduction potential; SDF, sperm DNA fragmentation; TUNEL, terminal deoxynucleotidyl transferase dUTP nick-end labeling; SCSA, sperm chromatin structure assay; SCD, sperm chromatin dispersion assay.

Because no single biomarker reliably identifies relative Sertoli-cell FSH hyporesponsiveness, the FERTILE framework is proposed as a multidimensional conceptual framework designed to integrate biological factors that may influence effective FSH signaling and SDF. Rather than functioning as a numerical scoring system, which would require validated weighting and clinical cutoffs, the FERTILE framework is intended as a qualitative research stratification tool. The framework does not imply that all domains contribute equally in every patient or that abnormalities in one domain necessarily indicate impaired effective FSH signaling. Rather, it is intended to facilitate biological stratification, generate testable hypotheses, and support future prospective validation studies. The framework encompasses seven complementary domains (**Figure 1**):

− F (FSH & Effective Intratesticular Signaling): Circulating FSH concentrations and candidate evidence of FSH bioactivity and downstream intratesticular FSH-responsive signaling.
− E (Endocrine Markers): Inhibin B, anti-Müllerian hormone, and total and free testosterone, providing complementary information regarding Sertoli-cell activity, spermatogenic output, and androgen status.
− R (Receptor & Ligand Genetics): FSHB and FSHR variants and related genetic factors that may influence ligand production, receptor expression, or downstream signaling responsiveness.
− T (Testicular Structure): Testicular volume and structural findings identified through scrotal imaging or, where available, histopathological assessment.
− I (Inflammation & Oxidative Stress): Seminal ORP, leukocytospermia or pyospermia, genital-tract infection or inflammation, and clinical varicocele.
− L (Lifestyle & Environmental Factors): BMI and obesity, smoking, alcohol use, dietary habits, heat exposure, environmental toxins, occupational exposures, and other potentially modifiable factors.
− E (Evaluation of DNA Integrity): Quantitative assessment of SDF using validated assays such as TUNEL, SCSA, SCD, and Comet assay.

The proposed domains are supported by varying levels of clinical and experimental evidence and should therefore be interpreted collectively rather than individually. Some domains reflect established clinical parameters, whereas others represent candidate biological markers requiring further validation.

This qualitative framework encourages a comprehensive assessment across these seven complementary dimensions. By integrating these parameters, the FERTILE framework may facilitate biological stratification of male infertility, support the design of future prospective clinical studies, and guide validation of candidate biomarkers rather than immediate clinical decision-making. **Table 2** details the candidate clinical, laboratory, and research parameters and the biological relevance of each domain.

**Table 2:** The seven domains of the FERTILE framework.

Domain letter	Domain name	Candidate clinical, laboratory, and research parameters	Biological relevance within the proposed framework
F	FSH & Effective Intratesticular Signaling	Circulating serum FSH levels; candidate research measures of FSH bioactivity and downstream FSH-responsive intratesticular signaling, where available	Characterizes circulating endocrine stimulation and candidate evidence of effective intratesticular FSH action; serum FSH alone is not a direct measure of Sertoli-cell responsiveness.
E	Endocrine Markers	Serum Inhibin B, Anti-Müllerian Hormone (AMH), total and free testosterone	Provides complementary endocrine context reflecting Sertoli-cell activity, spermatogenic output, and androgen status.
R	Receptor & Ligand Genetics	Genotyping of FSHB promoter variants (e.g., −211G>T) and FSHR variants (e.g., −29G>A, p.T307A, and p.N680S)	Identifies genetic variants that may influence ligand production, receptor expression, or downstream signaling responsiveness.
T	Testicular Structure	Testicular-volume assessment; scrotal ultrasound and, where available, histopathological findings	Characterizes testicular size and structural context relevant to spermatogenic capacity.
I	Inflammation & Oxidative Stress	Seminal ORP; leukocytospermia or pyospermia; genital-tract infection or inflammation; clinical varicocele	Identifies inflammatory and oxidative contributors that may independently or interactively increase SDF.
L	Lifestyle & Environmental Factors	BMI and obesity; smoking; alcohol use; dietary habits; heat exposure; environmental toxins; occupational exposures	Identifies potentially modifiable exposures that may promote systemic or reproductive-tract oxidative stress and adversely affect sperm quality.
E	Evaluation of DNA Integrity	Quantitative SDF assessment using TUNEL, SCSA, SCD, or Comet assays	Characterizes the downstream DNA-integrity phenotype without identifying its underlying biological mechanism.

### 5.7 Clinical context and interplay with other hormones

Effective FSH signaling should be interpreted within the broader endocrine framework involving other hormonal regulators of spermatogenesis rather than as an isolated hormonal signal. LH stimulates Leydig cells to produce testosterone, which diffuses into the seminiferous tubules and acts primarily through androgen receptor signaling in Sertoli cells, thereby indirectly supporting germ-cell development and maturation (18). Subsequently, testosterone supports Sertoli-cell differentiation, tight junction formation, and blood–testis barrier integrity, and synergizes with FSH to promote germ-cell survival and maturation. Accordingly, the biological consequences of relative Sertoli-cell FSH hyporesponsiveness may depend partly on whether intratesticular androgen action is preserved or concurrently impaired, because FSH and androgen signaling provide complementary support for spermatogenesis (18, 20, 21). Hypogonadism due to insufficient LH or testosterone can impair spermatogenesis independently of FSH and contribute to adverse sperm quality and increased susceptibility to SDF (21, 59). Conversely, FSH therapy alone may be insufficient to fully optimize spermatogenesis or sperm genomic integrity when an underlying androgen deficit persists. Accordingly, the evaluation of male infertility should consider the entire hypothalamic-pituitary-gonadal (HPG) axis rather than isolated hormonal abnormalities (60, 61). Combination therapy with human chorionic gonadotropin (hCG) and FSH in men with hypogonadotropic hypogonadism may restore testosterone levels and improve spermatogenesis (21). Future research should examine how relative Sertoli-cell FSH hyporesponsiveness interacts with testosterone signaling and whether men with combined endocrine abnormalities derive differential benefit from tailored hormonal therapies.

### 5.8 SDF beyond FSH: varicocele, oxidative stress, and lifestyle factors

SDF is a multifactorial condition, and numerous established mechanisms contribute independently of effective FSH signaling. Varicocele, characterized by abnormally dilated veins of the pampiniform plexus, is one of the leading surgically correctable causes of male infertility. This pathology may promote testicular hyperthermia, oxidative stress, hypoxia, and impaired Sertoli-cell function, thereby contributing to increased SDF (62, 63). Surgical varicocelectomy in infertile men with clinical varicocele has consistently been associated with significant postoperative reductions in SDF. A meta-analysis of 11 studies involving 394 patients demonstrated a reduction in postoperative DNA fragmentation index (DFI) values, with a weighted mean difference (WMD) of −5.79% (95% CI: −7.39 to −4.19%) in the random-effects model and of −6.14% (95% CI: −6.90 to −5.37%) after sensitivity analysis (64). An earlier meta-analysis of 7 prospective studies involving 289 patients supported a similar reduction in DFI of -6.86% (95% CI: -10.04% to -3.69%) following varicocelectomy (62). The postoperative reduction in SDF appears time-dependent: pooled analyses showed a mean difference of −6.74% (95% CI: −9.40% to −4.08%) at 3 months after surgery and a greater reduction of −12.39% (95% CI: −22.41% to −2.36%) at 6 months (65). Additionally, a systematic review and meta-analysis of 29 studies involving 1,491 men confirmed a significant reduction in SDF post-varicocelectomy, reporting a standardized mean difference (SMD) of −1.125 (95% CI: −1.410 to −0.840; p < 0.0001) (63). Collectively, these analyses underscore the therapeutic benefit of varicocele repair in lowering sperm DNA damage. These findings emphasize that established causes of SDF should be identified and managed before considering additional hypothesis-driven mechanisms such as relative Sertoli-cell FSH hyporesponsiveness. Another factor associated with SDF is oxidative stress, which may induce DNA strand breaks, oxidative base modifications, and chromatin cross-linking, thereby compromising sperm DNA integrity (66). Several randomized and prospective studies have evaluated antioxidant regimens including vitamin C, vitamin E, coenzyme Q10, N-acetylcysteine, L-carnitine, and combination therapies, as summarized in recent systematic reviews and meta-analyses (65, 67). Although several studies have reported modest reductions in SDF and improvements in selected semen parameters, the overall evidence remains heterogeneous, and the magnitude of clinical benefit continues to be debated (65). Lifestyle modifications, including smoking cessation, weight optimization, and regular physical activity, may also reduce oxidative stress and improve sperm quality (65). For example, in a prospective study of 105 obese men, participation in a 12-week lifestyle intervention consisting of dietary modification and daily exercise was associated with a significant reduction in SDF, with mean DFI decreasing from 20.2% to 17.5% (p < 0.001) (68). Accordingly, optimization of reversible causes of oxidative stress and other established contributors to SDF should remain a fundamental component of clinical management, irrespective of the proposed hypothesis regarding effective FSH signaling.

## 6 Research and translational framework

The proposed hypothesis identifies several priorities for future translational research aimed at determining whether relative Sertoli-cell FSH hyporesponsiveness contributes to SDF. The following sections outline experimental models, candidate biomarkers, and clinical research strategies that may facilitate systematic validation of the proposed hypothesis and its potential translation into clinical practice.

### 6.1 *In vitro* and ex vivo experimental models

Future research should prioritize the development of experimental approaches capable of assessing relative Sertoli-cell FSH hyporesponsiveness. These approaches should also investigate how variation in effective FSH signaling influences chromatin remodeling, physiological DNA-break repair, apoptotic regulation, and other Sertoli-cell functions involved in germ-cell development and quality control. Where ethically and technically feasible, ex vivo human seminiferous tubule cultures or related experimental systems may allow controlled manipulation of FSH exposure and assessment of downstream Sertoli-cell responses. Potential readouts could include caspase activation, DNA fragmentation, chromatin packaging, protamination status, oxidative stress markers, and other indicators of Sertoli-cell support for germ-cell development (13, 14, 16). Complementary animal models could employ FSHR knockdown, overexpression, knock-in polymorphisms, or conditional Sertoli-cell-specific genetic manipulation to clarify dose-response relationships and identify downstream pathways regulated by FSH (26, 58). Such models may also help determine whether alterations in FSH signaling influence spermatogenesis and downstream processes involved in chromatin remodeling and sperm DNA integrity (13, 35). These experiments should distinguish Sertoli-cell-mediated effects from direct germ-cell effects by employing co-culture systems, conditional knockouts, or targeted pharmacologic interventions. Demonstrating that experimentally induced alterations in effective FSH signaling reproduce patterns of sperm DNA damage observed in clinical settings would provide important biological support for the proposed hypothesis.

### 6.2 Human phenotyping and biomarker-integrated cohort studies

Future prospective human studies should consist of comprehensive endocrine profiling, genetic stratification, standardized SDF testing, and, where ethically permissible, testicular tissue analysis. Men with elevated SDF and otherwise normal conventional semen parameters should be of particular interest because they align with the clinical pattern that the proposed model seeks to address. Assessment should include FSH, LH, testosterone, estradiol, inhibin B, AMH, testicular volume, conventional semen parameters, SDF assay type and threshold, and relevant clinical factors. FSHB and FSHR genotyping should be incorporated as stratification variables rather than deterministic markers (32). When available in research settings, testicular tissue could be explored for the assessment of FSHR expression and downstream FSH-responsive signaling pathways within Sertoli cells (22, 58). Correlation of these data with SDF severity, protamination defects, oxidative stress indices, and reproductive outcomes could provide important evidence supporting or refuting the existence of a clinically meaningful phenotype of relative Sertoli-cell FSH hyporesponsiveness.

### 6.3 Pharmacogenetically stratified FSH trials

Future randomized clinical trials evaluating FSH therapy for elevated SDF should adopt biologically stratified designs rather than treating all men with increased SDF as a homogeneous group. Patients should be classified at baseline according to SDF level, FSHB/FSHR genotype, serum FSH, inhibin B, testicular volume, and other markers of Sertoli-cell function. Primary biological endpoints could include absolute and percentage change in SDF, protamination status, oxidative stress markers, and conventional semen parameters (14, 35). Secondary clinical endpoints should encompass fertilization rate, embryo development, clinical pregnancy, miscarriage, live birth, and time to conception. Trials should be adequately powered to evaluate genotype–treatment interactions rather than relying on *post hoc* subgroup analyses (36). Such studies could determine whether FSH therapy is more effective in biologically defined subgroups with features suggestive of relative Sertoli-cell FSH hyporesponsiveness rather than being applied empirically to all men with elevated SDF. Importantly, treatment effects should be assessed over biologically appropriate spermatogenic intervals. In varicocelectomy studies, reductions in SDF have been observed at 3 months and may continue to improve through 6 months, suggesting that follow-up extending beyond a single spermatogenic cycle may be necessary when evaluating interventions targeting sperm DNA integrity (65).

### 6.4 Combined therapeutic approaches and outcome standardization

The multifactorial nature of SDF provides a biological rationale for investigating combination therapeutic approaches rather than relying exclusively on single interventions. Elevated SDF may arise from defects in chromatin remodeling and apoptotic quality-control mechanisms within the testes, but it may also result from oxidative stress associated with conditions such as varicocele, inflammation, unhealthy lifestyle, and hazardous environmental exposures (12, 13, 64). Thus, future research should explore whether biologically sensible combinations such as FSH therapy plus varicocele repair, antioxidant strategies, lifestyle modification, or targeted ART laboratory techniques act synergistically in defined subgroups. Recent meta-analyses indicate that varicocelectomy produces significant reductions in SDF over three to six months, while FSH therapy may achieve meaningful SDF reductions in selected cohorts (35, 65). Matching SDF-directed interventions to the predominant biological mechanisms contributing to SDF may improve treatment outcomes compared with a uniform treatment approach. In addition, progress in the management of SDF depends on the standardization of SDF assessment. Current investigations use multiple methods to determine SDF, including TUNEL, SCSA, SCD, and Comet assay, all assessing overlapping yet unique characteristics of DNA damage and providing non-comparable absolute numbers (2). Future research needs to specify the method applied in SDF quantification, use threshold values, and report absolute and percentage improvements as well as criteria for a successful response. Without greater methodological standardization, comparison across studies and translation of research findings into clinical practice will remain challenging.

### 6.5 Additional research directions: retinoic acid and metabolic cross-talk

Retinoic acid, a derivative of vitamin A, is essential for spermatogonial differentiation, meiotic initiation, and coordination of the seminiferous epithelial cycle (41, 69). Sertoli cells participate in retinoid metabolism and control the local concentration of retinoic acid within the seminiferous epithelium. Several experimental studies suggest an interaction between FSH signaling and retinoic acid pathways, notably through FSH-regulated expression of retinoic acid-synthesizing enzymes and binding proteins, although the precise molecular mechanisms remain incompletely characterized. This has led to the suggestion that disruption of FSH signaling may disturb the intratesticular retinoic acid balance and thereby impair spermatogonial differentiation and meiotic entry (41). Reduced retinoic acid signaling may impair spermatogonial differentiation and meiotic initiation and could contribute indirectly to downstream abnormalities in spermatogenesis and sperm maturation (69, 70). Future studies should investigate the interaction between FSH signaling and retinoid pathways and determine whether alterations in retinoic acid signaling contribute to the biological mechanisms underlying sperm DNA damage in subsets of infertile men. In addition, metabolic cross-talk between Sertoli cells and germ cells, particularly lactate dynamics, glucose utilization, and lipid metabolism, warrants further investigation. Emerging evidence suggests that Sertoli cells may adapt their metabolic phenotype in response to FSH and androgen signaling, thereby altering nutrient utilization and metabolic support of developing germ cells (28, 29). Although direct evidence linking altered Sertoli-cell metabolism to SDF remains limited, understanding how these metabolic interactions influence sperm nuclear integrity could uncover novel therapeutic targets.

### 6.6 Impact of SDF on reproductive outcomes and relevance to FSH responsiveness

Multiple systematic reviews and meta-analyses have evaluated the association between SDF and reproductive outcomes across natural conception and assisted reproductive technologies. A recent meta-analysis including more than 12,000 IVF and ICSI cycles reported that elevated SDF was associated with reduced implantation and pregnancy rates, particularly in conventional IVF, and showed a detrimental trend for live birth outcomes (7). Another comprehensive review suggested that elevated SDF may have a greater detrimental impact on conventional IVF than on ICSI outcomes, a difference hypothesized to reflect oocyte-mediated DNA repair capacity and other biological factors (1, 8). These findings underscore the clinical importance of identifying and addressing the biological mechanisms contributing to elevated SDF. Within the context of the present hypothesis, it remains unknown whether variation in effective FSH signaling contributes to these adverse reproductive associations in a subset of infertile men. If future studies demonstrate that relative Sertoli-cell FSH hyporesponsiveness contributes to persistent sperm DNA damage or defective germ-cell quality-control mechanisms, biologically targeted interventions could potentially improve semen quality and reproductive outcomes in appropriately selected patients.

### 6.7 Individualized patient selection and the APHRODITE criteria

The APHRODITE criteria, recently proposed by an expert panel, advocate for a more individualized selection of male patients with idiopathic infertility who may benefit from hormonal treatment. These criteria integrate clinical history, endocrine profiles, particularly testosterone and FSH levels, and semen parameters to identify men likely to have testicular dysfunction responsive to hormonal manipulation (71). According to these criteria, patients are classified into five distinct groups:

− Group 1: Congenital or acquired hypogonadotropic hypogonadism, characterized by low FSH, low LH, and reduced total testosterone, usually combined with azoospermia or severe oligozoospermia.
− Group 2: Idiopathic male infertility with impaired conventional semen parameters, normal serum FSH, and normal serum total testosterone concentrations.
− Group 3: A hypogonadal state (biochemical hypogonadism) with lowered semen parameters, normal FSH, and reduced total testosterone.
− Group 4: Hypergonadotropic hypogonadism with lowered semen parameters, elevated FSH, and normal or reduced testosterone.
− Group 5: Unexplained male infertility in the context of unexplained couple infertility, characterized by normal conventional semen parameters, normal FSH, and normal testosterone.

Within this framework, relative Sertoli-cell FSH hyporesponsiveness could represent an additional layer of biological stratification. Men meeting APHRODITE criteria, particularly those classified as Group 2 or Group 5, who also exhibit genetic or endocrine features potentially associated with altered effective FSH signaling (for example, specific FSHR p.N680S genotype groups), may represent appropriate candidates for future pharmacogenetically stratified FSH trials (34, 71). If prospectively validated, incorporation of the proposed hypothesis into the APHRODITE criteria could further refine biological patient stratification and optimize the selection of candidates for hormonal therapy. Until such evidence is available, clinical management should remain guided by established evidence-based recommendations, while the proposed framework should be considered a research tool for future investigation.

### 6.8 Biological validation and hypothesis testing

To facilitate rigorous scientific evaluation, the proposed FERTILE framework should define how its central hypothesis could be falsified and outline the minimum dataset required for future prospective validation. The proposed hypothesis of relative Sertoli-cell FSH hyporesponsiveness would be considered unsupported if the following observations were consistently demonstrated:

1. Prospectively stratified randomized trials demonstrate that recombinant FSH therapy does not improve SDF, chromatin packaging, or related biological endpoints in predefined pharmacogenetic subgroups compared with appropriate controls.
2. *In vitro* models of human seminiferous tubules show that blocking downstream FSHR signaling cascades (such as the cAMP/PKA or PI3K/Akt pathways) does not affect protamine incorporation, chromatin-packaging markers and DNA integrity-related endpoints.
3. *In vivo* studies demonstrate that variation in FSHR signaling efficiency does not correlate with changes in established markers of Sertoli-cell support, metabolism, or germ-cell survival.

To validate this framework, future prospective clinical studies should incorporate a standardized minimum dataset, including:

− Endocrine Profiling: Morning fasting serum FSH, LH, total and free testosterone, estradiol, sex hormone-binding globulin (SHBG), Inhibin B, and Anti-Müllerian Hormone (AMH) (39, 54).
− Genetic and Pharmacogenetic Characterization: Genotyping of key FSHB promoter variants (e.g., −211G>T) and FSHR polymorphisms (e.g., p.N680S and p.T307A) (32–34).
− Standardized SDF assessment using validated assays such as TUNEL and/or SCSA, with predefined thresholds and reporting standards (2, 35).
− Chromatin Compaction Metrics: Measurement of the Protamine 1-to-protamine 2 ratio (P1/P2) and the proportion of spermatozoa with histone retention detected by aniline blue staining (13, 14).
− Clinical and Environmental Confounder Tracking: Testicular volume, presence and grade of varicoceles, seminal ORP, smoking history, BMI, and occupational exposures (2, 12).

## 7 Limitations and provisional nature of the proposed model

First, there is currently no direct evidence demonstrating that relative Sertoli-cell FSH hyporesponsiveness causes elevated SDF in men. The proposed model integrates disparate lines of clinical, experimental, and pharmacogenetic evidence relating FSH signaling to Sertoli-cell function, pharmacogenetic associations, and clinical responses to FSH therapy without direct assessment of Sertoli-cell FSH responsiveness in human tissue. Validation would require the assessment of FSHR abundance, receptor localization, ligand-induced signaling activity, and downstream phospho-signaling responses in Sertoli cells obtained from men with and without elevated SDF, correlated with standardized SDF assays, chromatin-packaging markers, endocrine profiles, genotype, and reproductive outcomes. Such comprehensive datasets are currently unavailable.

Second, SDF is a multifactorial phenotype. Relative Sertoli-cell FSH hyporesponsiveness, if validated, would likely represent one modifier of SDF severity rather than a universal explanation. Elevated SDF may result from varicocele, infection, inflammation, leukocytospermia, oxidative stress, obesity, aging, heat exposure, toxicants, smoking, lifestyle factors, or post-testicular oxidative damage acquired during epididymal transit and sperm storage (2). Over-attributing SDF to impaired FSH responsiveness could lead to misclassification and inappropriate therapy, underscoring the need for a comprehensive evaluation of contributory factors.

Third, the interpretation of available clinical literature is limited by methodological heterogeneity. Studies evaluating SDF differ in assay type, thresholds, laboratory protocols, patient selection, and outcome reporting. The principal SDF assays, including TUNEL, SCSA, SCD, and Comet assay, measure overlapping but non-identical aspects of sperm DNA damage. The standardization of assay selection, calibration, and reporting is essential for the meaningful comparison of studies and translation into practice (2, 7). Moreover, pharmacogenetic evidence remains promising but clinically incomplete, as genotype-associated effects are generally modest and appear to be influenced by additional genetic, biological, environmental, and lifestyle factors (32). Current evidence does not support the routine use of genetic polymorphisms as stand-alone tools for therapeutic decision-making in male infertility, emphasizing the need for integrated clinical assessment. The hypothesis presented here is intended to stimulate research and refine patient stratification rather than to support widespread off-label FSH use. Accordingly, the proposed framework should be regarded as a hypothesis-generating conceptual model whose clinical utility remains to be established through prospective translational research.

## 8 Conclusion

Relative Sertoli-cell FSH hyporesponsiveness may represent an under-recognized biological modifier of SDF. By proposing that serum FSH concentration alone does not fully reflect effective intratesticular FSH action, this review integrates evidence from FSHR biology, pharmacogenetics, mechanistic models, and clinical studies. The proposed hypothesis suggests that relative Sertoli-cell FSH hyporesponsiveness could influence chromatin remodeling, physiological DNA-repair coordination, apoptotic quality control, and metabolic or redox support during spermiogenesis, thereby increasing susceptibility to persistent sperm DNA damage. This model may explain why some men with apparently preserved endocrine profiles and conventional semen parameters nonetheless exhibit elevated SDF and impaired reproductive outcomes.

However, the hypothesis remains provisional; its translational value depends on prospective studies combining standardized SDF assays, endocrine profiling, FSHB/FSHR genotyping, direct or indirect Sertoli-cell functional readouts, and clinically meaningful reproductive endpoints. Until such evidence becomes available, the proposed FERTILE framework and the concept of relative Sertoli-cell FSH hyporesponsiveness should be regarded as hypothesis-generating research tools intended to facilitate biological stratification and future translational investigation rather than as a basis for changing routine evidence-based clinical practice.

## Data Availability

No datasets were generated or analyzed for this study. The original theoretical framework and hypotheses are contained within the article.
